# A pilot randomized trial comparing face-to-face and internet-based unified transdiagnostic treatment for adolescents with anxiety and depressive disorders in Iran

**DOI:** 10.3389/fpsyt.2025.1500214

**Published:** 2025-08-18

**Authors:** Nasim Mousavi, Fereshteh Momeni, Abbas Pourshahbaz, Hamid Poursharifi, Zahra Daneshian, Gerhard Andersson

**Affiliations:** ^1^ Department of Clinical Psychology, University of Social Welfare and Rehabilitation Sciences, Tehran, Iran; ^2^ Shahid Beheshti University, Tehran, Iran; ^3^ Department of Behavioral Sciences and Learning, Linköping University, Linköping, Sweden; ^4^ Department of Biomedical and Clinical Sciences, Linköping University, Linköping, Sweden

**Keywords:** adolescents, anxiety disorders, depressive disorders, transdiagnostic treatment, internet-based treatment

## Abstract

**Objective:**

The use of internet-based treatments has increased significantly in recent years. As remote technologies continue to evolve, psychotherapy research is progressively shifting toward these approaches. Anxiety and depressive disorders are highly prevalent in adolescents, imposing significant personal and societal costs. Identifying effective and scalable treatments for this age group is therefore essential. This study aimed to compare the efficacy of face-to-face and internet-based unified transdiagnostic treatment in reducing symptoms and improving functioning in adolescents with anxiety and depressive disorders.

**Methods:**

A pilot randomized controlled trial (RCT) with pre-test, post-test, and follow-up assessments was conducted to compare the efficacy of face-to-face and internet-based treatments. Forty-nine adolescents (aged 13–18 years) from Tehran, Alborz, Gilan, and Kerman were randomly assigned to one of three groups*: face-to-face treatment, internet-based treatment, or a control group*. Assessments were conducted before treatment, after treatment, and at six months post-treatment. A Mixed-model Analysis of Variance was used for data analysis.

**Results:**

Both face-to-face and internet-based transdiagnostic treatments demonstrated similar efficacy in reducing anxiety and depression symptoms, improving functioning, decreasing negative affect, and reducing avoidance in adolescents. However, neither treatment significantly improved positive affect or distress tolerance. Additionally, the effects in the internet-based group were maintained through the follow-up phase, while the face-to-face treatment group did not sustain these results by the six-month follow-up.

**Conclusion:**

Based on the results of this study, internet-based transdiagnostic treatment is also a viable option for treating anxiety and depressive disorders. The large scale implementation of internet-based transdiagnostic psychotherapy could be an effective strategy to bridge the significant gap between adolescents’ mental health needs and the availability of evidence-based treatments for anxiety and depression.

**Clinical trial registration:**

https://irct.behdasht.gov.ir/trial/62220, identifier IRCT20220226054129N1.

## 1 Introduction

Adolescence is a period characterized by increased vulnerability to mental disorders and the emergence of mental health issues, including anxiety and depression (1, 2). National research on the prevalence of psychiatric disorders among children and adolescents in Iran (3) indicate that 14.1% of individuals aged 6 to 18 suffer from an anxiety disorder, while 2.2% are affected by a mood disorder. These adolescents are at a higher risk of future mental health problems, academic difficulties, challenges in peer relationships, suicide, and substance use compared to their peers (4, 5). These disorders impose significant societal costs, including increased reliance on public services and decreased workforce participation. These disorders are typically chronic, have a high risk of relapse, and present a low likelihood of recovery without treatment (6, 7). A major challenge in treating these disorders is the high comorbidity of anxiety and depression, which is particularly prevalent during adolescence (8) and increases compared to childhood (9). In Iran, the comorbidity rate of these disorders among individuals aged 6 to 18 years is 51.1% (3).

Research on common higher-order factors underlying anxiety disorders, depressive disorders, and other emotional disorders suggests that increased activity in neural structures related to negative affect, deficits in emotion regulation, and neuroticism are present across all these conditions (10–12). Several structural models have been proposed to explain emotional disorders, with treatment protocols based on these models primarily focusing on cognitive-behavioral approaches tailored to each disorder. However, interventions targeting multiple disorders simultaneously focus on shared mechanisms and adapt treatment to the unique comorbidities of each patient. These are known as transdiagnostic interventions (13, 14). Among these, Barlow et al. (2017) developed the “Unified Protocol for Transdiagnostic Treatment of Emotional Disorders (UP),” which applies to all anxiety and depressive disorders, as well as other conditions with strong emotional components (15). The transdiagnostic protocol for adolescents is designed to address a wide range of emotional disorder symptoms in individuals aged 13 to 18 years. This protocol incorporates evidence-based techniques, including preventing emotional avoidance, enhancing cognitive flexibility, and modifying maladaptive action tendencies (12).

A major challenge in this field is the unequal distribution of mental health facilities and the concentration of specialists in large cities. On average, there are nine mental health specialists per 100,000 people worldwide, and Iran falls significantly below this standard (16). According to 2016 statistics on household information technology usage in Iran, 77.9% of urban households and 57% of rural households have Internet access, and 35.8% of individuals use the Internet to search for health information (17). Due to its large demographic area and significant number of underserved regions, Iran has substantial potential for utilizing Internet-based health technology (18).

Studies suggest that Internet-based psychological treatments can be a promising solution for increasing access to evidence-based treatments and are comparable to face-to-face treatment in terms of effectiveness (19). However, implementing these interventions in adolescents remains challenging, and there are issues noted in research such as lack of evidence on efficacy, high dropout rates, limited effectiveness in preventing relapse, the absence of lasting therapeutic effects, reduced therapist control over the treatment environment, difficulties in empathy, challenges in fostering therapeutic motivation, concerns about data confidentiality, ethical and legal considerations, concerns related to privacy such as the possibility of eavesdropping during therapy sessions at home, and effective for depression but less effective for anxiety (20–28).

Studies in Iran on the efficacy of Internet-based psychotherapy for reducing symptoms of anxiety and depression in adolescents suggest that this treatment is effective. However, these studies has several limitations, including small sample sizes, absence of control groups, and lack of clinical patient’s inclusion (29–34).

Internet-based psychological therapies are increasingly used, but their efficacy and implementation for adolescents remain challenging in mental health care. Among various Internet-based therapies, guided therapy delivered through a website via videos, images, and writing assignments was chosen for this study. This study aims to compare the efficacy of face-to-face and Internet-based unified transdiagnostic treatments in reducing symptoms and improving the adolescents functioning in anxiety and depressive disorders. Accordingly, the following primary research questions were formulated:

1. Which is more effective in reducing symptoms of depression and anxiety in adolescents: face-to-face or Internet-based treatment?
2. Which treatment is more effective in improving adolescent functioning: face-to-face or Internet-based transdiagnostic treatment?

## 2 Materials and methods

### 2.1 Study design

This study employed a pilot randomized 2 X 3 experimental design with a pre-test and post-test within-group factor, and a between-group factor with two experimental groups and one control group. A six-month follow-up was also conducted. This study followed the CONSORT (Consolidated Standards of Reporting Trials) guidelines. The CONSORT checklist was used to ensure comprehensive and transparent reporting of the trial’s methodology and results (35). The checklist is provided in the Supplementary Materials.

### 2.2 Procedure

**Figure 1:**
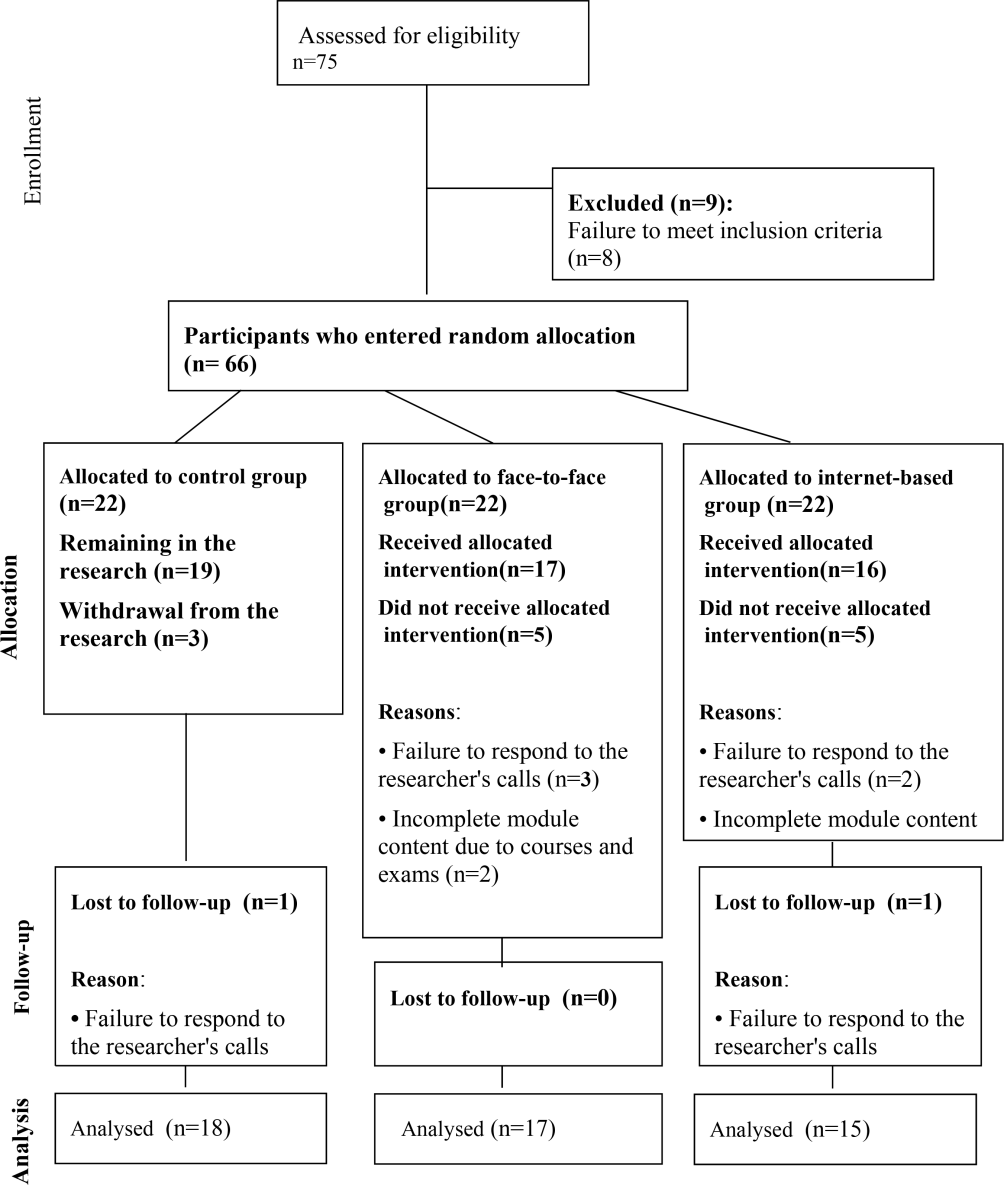
CONSORT diagram. This flow diagram was adapted from the CONSORT 2010 statement: Schulz KF, Altman DG, Moher D, for the CONSORT Group. CONSORT 2010 statement: updated guidlines for reporting parallel group randomised trials. BMJ. 2010;340:c332.

Participants were recruited through posters distributed online, in psychology clinics, schools, and via therapists. Adolescents with anxiety and depressive disorders residing in *Tehran, Alborz, Gilan and Kerman* were invited to participate. Adolescents who responded to the invitations underwent a structured clinical interview (SCID-5) via phone, conducted by a clinical psychologist. The study commenced in the summer of 2022, with a total of 75 individuals responding to the invitation. The planned sample size was 22 participants per group, determined. The sample size was determined based on previous research and Cochran’s formula (36) with an alpha level (α) of 0.05, a beta (β) of 0.20 (power = 80%), and an assumed attrition rate of 20%. The calculation was based on a medium effect size (d = 0.5), as supported by previous studies in similar interventions. The software used for the calculation was SPSS 2 (37–40). The inclusion criteria were as follows: 1. Age between 13 and 18 years. 2. Residence in one of the specified provinces. 3. Diagnosis of depressive and/or anxiety disorder (comorbidity with other psychiatric conditions such as disruptive behavior disorder, attention deficit hyperactivity disorder (ADHD), eating disorders, and substance use was acceptable if these did not interfere with treatment, provided the primary diagnosis was anxiety or depression). 4. No prior participation in a CBT course. 5. If taking medication, a stable dose must have been maintained for at least three months for SSRIs and one month prior for benzodiazepines.6. Access to a computer or mobile phone with a high-speed internet connection. The exclusion criteria included comorbid bipolar or psychotic disorders, autism spectrum disorder, suicidal tendencies, and recent psychiatric hospitalization. Additional exclusion criteria were as follows: 1. Drug or alcohol abuse if it interfered with treatment. 2. Participation in another treatment during the research period. 3. Absence from treatment sessions for more than two consecutive sessions, or failure to submit worksheets and follow up on treatment in the Internet-based group for more than one month. Nine adolescents did not meet the inclusion criteria and were excluded. A total of 66 individuals were included and randomly assigned to one of three groups. Participants were randomly assigned to three groups using the “random allocation rule” (41) by the researchers. An independent researcher generated the randomization list using computer-generated random numbers. Another researcher, who was not involved in participant recruitment, conducted the randomization. Written informed consent was obtained from each adolescent and one of their parents. **Figure 1** presents the attrition diagram based on CONSORT (42) along with the reasons for attrition.

Participants completed the demographic information form and questionnaires during the pre-test phase. Before data processing, researchers ensured that there were no significant differences in gender, age, or city of residence among the groups. One of the authors (N.M.) completed an 8-hour online training course led by Dr. Jill Ehrenreich-May (the primary protocol developer) before initiating the treatment.

### 2.3 Measures

#### 2.3.1 Structured clinical interview for DSM-5 disorders

This is a semi-structured interview designed for diagnosing major DSM-5 disorders. While its coverage and language is primarily intended for individuals over 18 years old, it can also be adapted for adolescents by modifying the wording of certain questions (43). SCID-5 has demonstrated substantial to excellent interrater reliability for most major psychiatric disorders (43–45). A meta-analysis of SCID versions reported kappa values ranging from 0.61 to 0.81, reflecting good to excellent agreement between raters (46). Studies have shown moderate to high test-retest reliability, with kappa values typically ranging from 0.56 to 0.78, depending on the disorder assessed (47). In studies on Iranian populations, the kappa coefficient for depressive disorders was 0.69, while for anxiety disorders, it was 0.34. In studies outside Iran, the Kappa coefficient for test-retest reliability in depressive disorders was 0.69, and for anxiety disorders, it was 0.71 (43, 48, 49).

#### 2.3.2 Revised children’s anxiety and depression scale

The RCADS was used to assess anxiety and depression in adolescents. This is a 47-item self-report scale that comprising six subscales: separation anxiety, social anxiety, generalized anxiety, obsessive-compulsive disorder, panic disorder, and major depression. The first five subscales are added together to obtain a total anxiety score. Items are rated on a 4-point Likert scale ranging from 0 (strongly disagree) to 3 (Strongly agree). The RCADS has demonstrated strong internal consistency (α > 0.80) and good convergent validity with other measures of anxiety and depression (50). The Persian version of the RCADS also exhibits good internal consistency, with Cronbach’s alpha values indicating high reliability. Exploratory factor analysis (EFA) identified a six-factor structure explaining 55.3% of the scale variance, which was confirmed by confirmatory factor analysis (CFA) (51).

#### 2.3.3 Positive and negative affect schedule

The PANAS is a 20-item self-report instrument that includes two mood-related subscales: Positive Affect (PA) and Negative Affect (NA). This Schedule assesses these subscales as orthogonal dimensions on a five-point Likert scale ranging from 1 to 5. The PANAS has demonstrated excellent internal consistency, with Cronbach’s alpha ranging from 0.86 to 0.90 for Positive Affect (PA) and 0.84 to 0.87 for Negative Affect (NA). Over an eight-week period, the test-retest reliability was 0.68 for PA and 0.71 for NA, indicating moderate stability over time. The two subscales are relatively independent, with correlations typically around -0.10 to -0.23, supporting their distinctiveness (52). In Iran, *Bakhshipoor* and *Dejhkam* (2006) investigated the two-factor structure of this instrument, reporting validity estimates with standardized factor loadings of 0.87 for Positive Affect and 0.85 for Negative Affect. The internal consistency coefficients for the two subscales for both subscales were 0.87 (53).

#### 2.3.4 Emotional avoidance strategy inventory for adolescents

The EASI-A was used to assess emotional avoidance in adolescents. This 33-item self-report inventory records responses on a Likert scale from 0 (Not at All True of Me) to 4 (Extremely True of Me). Factor analysis identified three factors: “Avoidance of Thoughts and Feelings”, “Avoidance of Emotional Expression” and “Distraction” (54). The EASI-A has demonstrated good predictive validity and reliability in samples of children and adolescents, with high convergent validity (r = 0.52) and strong incremental validity (54). The Persian version of this inventory was validated by the researchers of the present study and demonstrated strong validity. The internal consistency reliability of the Persian version, measured using Cronbach’s alpha, was reported 0.71 (55).

#### 2.3.5 Distress tolerance scale

The DTS assesses an individuals’ ability to tolerate distressing emotions and consists of 15 items. Responses are rated on a five-point Likert scale ranging from 1(Strongly Agree) to 5 (Strongly Disagree). *Simons* and *Gaher*, the developers of the scale, reported Cronbach’s alpha coefficients of 0.72, 0.82 and 0.70 for the subscales, and 0.82 for the total scale (56). In Iran, Cronbach’s alpha coefficients for the Tolerance, Absorption, Evaluation, and Regulation subscales were 0.75, 0.77, 0.70, and 0.75, respectively. The test-retest correlation coefficients over a two-month interval for these subscales and the total scale were 0.71, 0.69, 0.77, 0.73, and 0.79, respectively, all statistically significant (57).

#### 2.3.6 Adolescent life interference scale

The ALIS-I was used to assess adolescent functioning. Developed by *Schniering* et al., is the first scale designed to measure functional deficits related to anxiety and depression symptoms specifically in adolescents (ages 11 to 18 years). The ALIS-I consists of 26 items across four subscales: withdrawal/avoidance, Somatic Symptoms, Problems with Study/Work, and Peer Problems. Items are rated on a five-point Likert scale from 0 (Strongly Disagree) to 4 (Strongly Agree). Internal consistency has been reported as good, with values ​​ranging from 0.76 for the Somatic Symptoms subscale to 0.94 for the total scale (9). In Iran, Cronbach’s alpha coefficients for the total scale and subscales ranged from 0.70 to 0.89. McDonald’s omega coefficients for the total scale and subscales were reported to be above 0.70. The test-retest reliability, measured using intra-class correlation coefficients, ranged from 0.80 to 0.92 for the total scale and subscales over a three-week period (58).

### 2.4 Intervention

#### 2.4.1 Face-to-face treatment

Before initiating the treatment process, the principal researcher (N.M.) trained one of the researchers (Z.D.) and two additional adolescent psychologists to assist with the implementation of face-to-face treatment. Sessions were conducted individually, held weekly for 45 minutes each, over 12 sessions. Participants in the face-to-face treatment group were distributed among four therapists. Worksheets were provided in printed or PDF format. A pamphlet based on UP-A for parent education was sent to one parent at the start of the treatment. Re-evaluations were conducted at the end of the treatment and again after six months.

#### 2.4.2 Internet-based treatment

**Figure 2:**
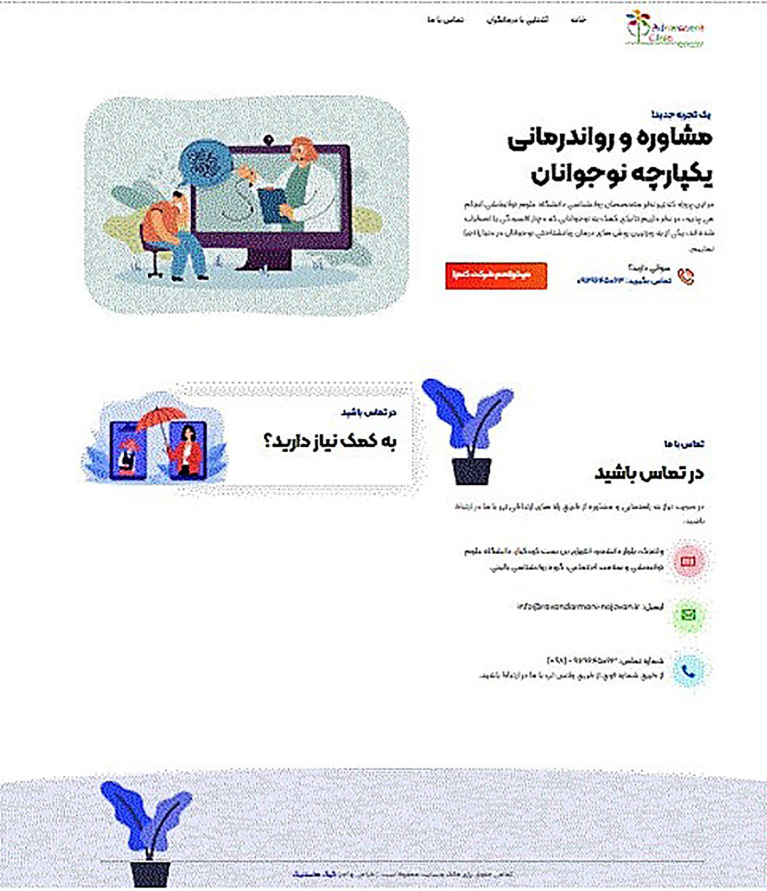
The entrance page of the website.

**Figure 3:**
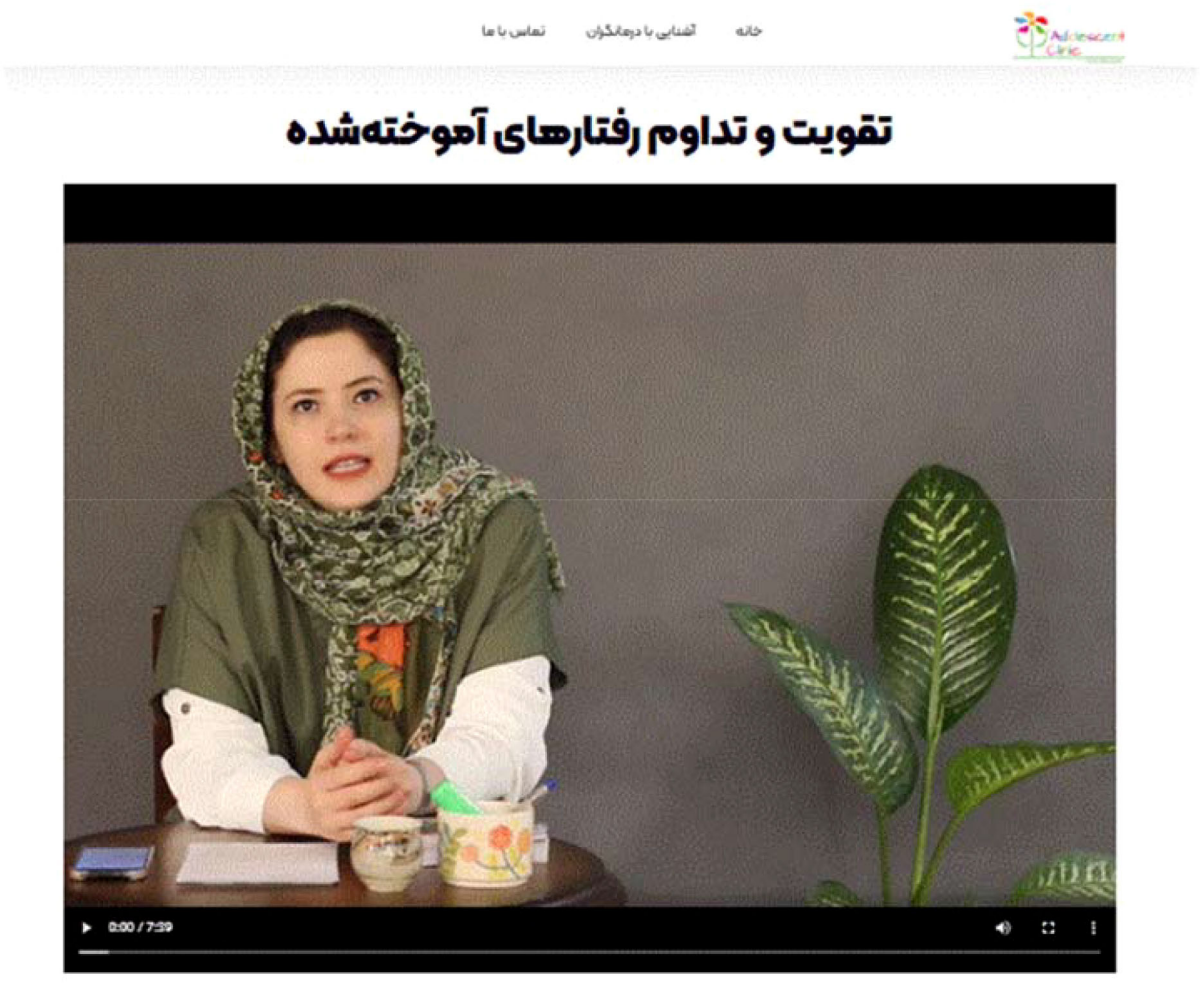
A view of one of the training videos.

**Figure 4:**
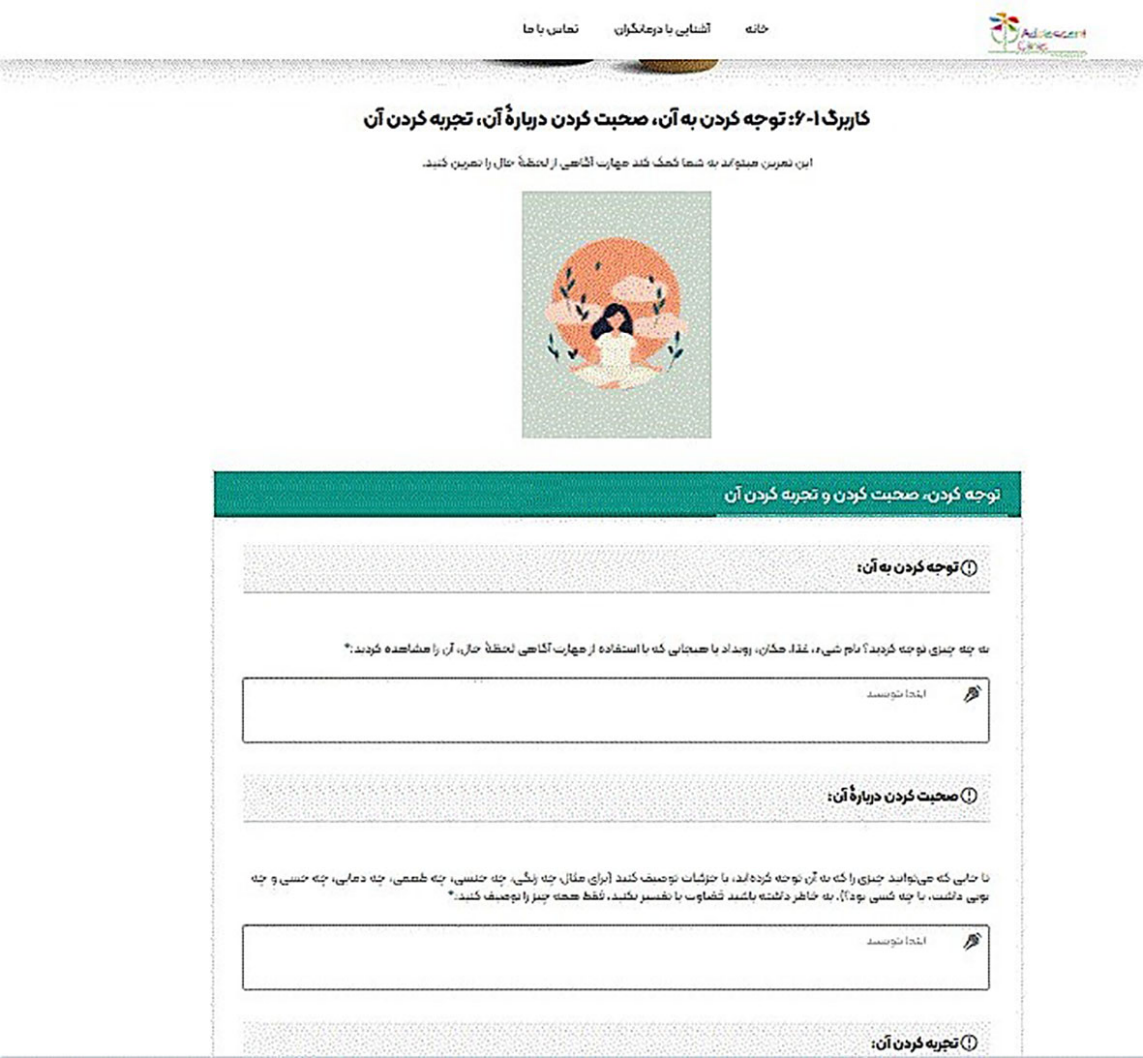
One of the worksheets.

A dedicated website (ravandarmani-nojavan.ir) was developed for this treatment, featuring therapeutic content. Participants registered on the site using their email addresses. The first part of the treatment was delivered via a link, which included videos, images, brochures, and a worksheet. Participants had one week to review the contents, complete the worksheet, and ask questions. They submitted the completed forms through the site, which were automatically forwarded to the therapist’s email. For security, a CAPTCHA was enabled for each worksheet. The principal investigator (N. M.) served as the sole therapist for Internet-based treatment participants. Participants could contact the therapist via voice or text messages on WhatsApp or Telegram for inquiries. Additionally, after completing each module, the therapist conducted a phone call to address questions and review material. A parental pamphlet was also provided. Upon completing module 7, participants received a brief performance report from the therapist. Follow-up evaluations were conducted at the end of the treatment and six months later. **Figures 2**–**4** display various aspects of the website.

The content of UP-A in face-to-face (12) and internet-based treatment (59) is listed in **Table 1**. In this study, no changes were made to the implementation of treatment protocols.

**Table 1:** Content of the unified protocol for adolescent emotional disorder (12) and Internet-based transdiagnostic treatment.

Module	Module title	Module goals	Number of sessions in face-to-face treatment	Educational materials in internet-based treatment	Time allocated to internet-based treatment
1	Building and Keeping Motivation	1: Orient the adolescent and family to treatment concepts and structure	1 or 2	-Video 1-1 -Educational Brochure -Video 2-1 -Worksheet 1-1: Identifying Key Problems	2 days
		2: Obtain three top problems from the adolescent, as well as severity ratings for each top problem			
2	Getting to Know Your Emotions and Behaviors	Goal 1: Begin learning emotion identification skills.	2 or 3	*Emotions I Have* (Worksheet 2.1)	1 day
		■ Goal 2: Provide education about emotions, their function, and their impact on behavior.		Video2-1 *Emotion Identification Practice* (Worksheet 2.2)	1 day
		■ Goal 3: Introduce the three parts of an emotional experience.		Video2-2 Educational Brochure Video2-3 Video2-4 Educational Brochure *Breaking Down My Emotions* (Worksheet 2.3)	3 days
		■ Goal 4: Discuss reinforcement and the maintenance of learned behavior		Video2-5 Educational Brochure *Tracking the Before, During, and After* (Form 2.1)	1 day
3	Introduction to Emotion-Focused Behavioral Experiments	1: Introduce the concepts of opposite action and emotion-focused behavioral experiments 2: Identify activities that the adolescent enjoys and can engage in during emotion-focused behavioral experiments.	1 or 2	Video 3-0 Video 3-1 Educational Brochure Video 3-2 *List of Commonly Enjoyed Activities* (Worksheet 3.1)	2 days
		3: Introduce the idea of tracking emotion and activity levels, and encourage the adolescent to conduct a behavioral experiment for sadness/withdrawal.		Video 3-3 Educational Brochure *Emotion and Activity Diary* (Worksheet 3.3) *Weekly Activity Planner* (Form 3.1)	2 days
4	Awareness of Physical Sensations	1: Describe the concept of physiological or body sensations and their relationship to intense emotions 2: Work with the adolescent to identify the feelings he has during emotional experiences using the body drawing and body scanning exercises	1 or 2	Educational Picture Video 4-1 Video 4-2 *Body Drawing* (Worksheet 4.1)	2 days
		3: Conduct sensational exposures with the client to promote awareness of body feelings.		Video 4-3 Educational table *Monitoring How My Body Feels* (Worksheet 4.2)	1 day
5	Being Flexible in Your Thinking	■ Goal 1: Introduce the concept of flexible thinking: automatic and alternative interpretations.	2 or 3	Video 5-1 Educational pictures	1 day
		■ Goal 2: Teach the adolescent the common “thinking traps.”		Video 5-2 Video 5-3 *Common Thinking Traps* (Worksheet 5.1)	2 days
		■ Goal 3: Introduce and ensure understanding of the Detective Thinking skill.		Video 5-4 *Evaluating My Thoughts Using Detective Questioning* (Worksheet 5.2) *Detective Thinking* (Form 5.1)	2 days
		■ Goal 4: Introduce and ensure understanding of the Problem Solving skill.		Video 5-5 Educational Brochure *Getting Unstuck—Steps for Solving a Problem* (Worksheet 5.4)	1 day
6	Awareness of Emotional Experiences	1: Introduce the rationale for present-moment awareness and practice present-moment awareness activities in session.	1 or 2	Video 6-1 Video 6-2 Educational Pictures *Notice it, Say Something About it, Experience it* (Worksheet 6.1)	2 days
		2: Introduce the rationale for nonjudgmental awareness and practice nonjudgmental awareness activities in session.		Video 6-3 Video 6-4 *Awareness Practice Monitoring* (Form 6.1) *Emotion Story* (Worksheet 6.2)	2 days
7	Situational Emotion Exposure	1: Review previously learned skills and create the adolescent’s *Emotional Behavior Form*	2+	Video 7-1 Educational Table *Emotional Behavior Form* (Form 7.1)	1 day
		2-Practice entering situations in which the adolescent has previously used maladaptive emotional behaviors, encouraging the adolescent to monitor her emotional reactions to these situations.		Video 7-2 Educational pictures Video 7-3 *My Emotion Ladder* (Form 7.3)	It depends on the adolescent ladder.
8	Reviewing Accomplishments and Looking Ahead	1: Review skills that have been most useful to the adolescent	1	*Skills I Know and How to Use Them* (Worksheet 8.1)	1 day
		2: Review progress		Patient Reading Report Submitted by Therapist Educational Brochure *Taking Stock of All I’ve Accomplished* (Worksheet 8.2)	2 days
		3: Make a post-treatment plan in order to prevent relapse		Video 8-1 *Becoming My Own Therapist!* (Worksheet 8.3)	1day

#### 2.4.3 Control group

The control group did not receive any intervention: however, participants and their parents could contact the therapist to ensure compliance with ethical guidelines. After the follow-up period, they were given the option to participate in either Internet-based or face-to-face treatment.

### 2.4 Data collection

Data were collected at three key time points: pretest (baseline), post-test (post-treatment), and follow-up. The specific measures taken at each point are summarized in **Table 2**.

**Table 2:** Measurement points.

Measurement Point	Description	Measures Taken
Pretest	Before the intervention starts	ALIS-I RCADS PANAS EASI-A DTS
Post-test	At the end of the intervention	ALIS-I RCADS PANAS EASI-A DTS
Follow-up	After 6 months post-intervention	ALIS-I RCADS PANAS EASI-A DTS

### 2.5 Outcomes

The primary endpoints of this study were the reduction of anxiety and depressive symptoms and the improvement of adolescents functioning. Reducing anxiety and depressive symptoms is crucial, as these mental health issues can significantly impact daily functioning, academic performance, and overall quality of life. Addressing these symptoms aligns with the primary goal of treatment: improving mental health outcomes in this vulnerable population (60, 61). Improvement in functioning_ including social, academic, and daily life activities_ was another key endpoint. This measure evaluates the practical impact of the intervention on adolescents’ lives, ensuring that the treatment provides tangible benefits (62). The secondary endpoints included reductions in emotional avoidance and negative affect, as well as improvements in distress tolerance and positive affect.

### 2.6 Ethics approval

This research was approved by the Ethics Committee of the University of Social Welfare and Rehabilitation Sciences under the ethical code IR.USWR.REC.1399.167, and registered as a clinical trial under the code IRCT20220226054129N1. To uphold ethical standards, informed consent was obtained, and participants were fully informed about the study and their right to withdraw at any time. Confidentiality principles were maintained, and participants’ data were securely archived.

### 2.7 Statistical analysis

Data analysis was conducted at both the descriptive and inferential levels. Descriptive analysis included frequency and mean calculations, while inferential analysis employed a mixed model 3 X 2 Analysis of Variance (ANOVA) with one between-group and one within-group factor. The mixed model ANOVA was deemed appropriate for this study as it accounts for both between-subject and within-subject variability over time. This approach allowed for the examination of main effects and interaction effect (63). The null hypothesis (H_0_) assumed that there are no significant differences between the groups in terms of reductions in anxiety and depression symptoms, functional improvement, or changes in avoidance and affect regulation. The alternative hypothesis (H_1_) posited that at least one treatment modality would lead to significantly different outcomes. A significant level (α) of 0.05 was set, and a two-way analysis was conducted to assess both within-group and between-group effects.

Prior to conducting ANOVA, its assumptions were assessed. To address the research questions, pairwise comparisons of three groups were performed across three measurement stages. Additionally, pairwise comparisons of the three measurement stages were conducted separately for each group. To control for Type I error due to multiple comparisons, Bonferroni adjustments were applied. Minimal missing data (<3%) were addressed using listwise deletion, as the proportion of missing values was insufficient to affect the results significantly and the same approach was applied to all outcomes. All statistical analysis were performed using SPSS 29.

## 3 Results

### 3.1 Participant recruitment

The final sample analyzed included 17 participants in the face-to-face treatment group, 15 in the internet-based treatment group, and 18 in the control group. **Table 3** presents the demographic characteristics of the participants. Statistical analysis using ANOVA or Chi-square tests (depending on the variable) indicated that the three groups did not significantly differ in terms of age (*p* = 0.06), gender (*p* = 0.85), residence in the provincial capital or other cities (*p* = 0.11), field of study (*p* = 0.88), or use of psychiatric medications (*p* = 0.89).

**Table 3:** Distribution of patients based on age range, gender, place of residence and drug use.

Characteristics	Groups			Test statistics	Level of significance
	Face-to-face	Internet-based	Control		
Average age (standard deviation)*	15.95 (1.63)	14.77 (1.76)	14.84 (1.21)	F=3.06	0.06
Girls, number (%)**	14 (82.35)	13 (86.67)	16 (88.89)	Value= 0.32	0.85
Residence, number (%)**				Value= 4.37	0.11
The provincial capitals^***^	16 (94.12)	11 (73.33)	17 (94.45)		
Other cities and towns	1 (0.06)	4 (26.67)	1 (0.06)		
Field of study, number (%)**				Value=2.41	0.88
Math and physics	2 (11.76)	1 (6.67)	0 (0)		
Experimental sciences	5 (29.41)	1 (6.67)	1 (5.56)		
Humanities	4 (22.53)	2 (13.33)	1 (5.56)		
Technical and vocational	3 (17.65)	0 (0)	1 (5.56)		
Before choosing a field	3 (17.65)	11 (73.33)	15 (83.33)		
Diagnoses (%)**				Value=13.14	0.67
Generalized Anxiety Disorder	1 (6.7)	3 (17.6)	2 (11.1)		
Social Anxiety Disorder	3 (20)	1 (5.9)	1 (5.6)		
Phobia	0	0	1 (5.6)		
Panic Disorder	1 (6.7)	1 (5.9)	1 (5.6)		
Other Anxiety Disorder	1 (6.7)	4 (23.5)	6 (33.3)		
Major Depressive Disorder	2 (13.3)	4 (23.5)	2 (11.1)		
Disruptive Mood Disorder	1 (6.7)	0	1 (5.6)		
Other Depressive Disorder	1 (6.7)	2 (11.8)	0		
Anxiety Disorders+ Depressive Disorders	5 (33.3)	2 (11.8)	4 (22.2)		
Comorbidities (%)**				Value=8.93	0.35
Obsessive-Compulsive Disorder	2 (13.3)	4 (23.5)	7 (38.9)		
Drug Abuse	0	1 (5.9)	0		
Misophonia	1 (6.7)	0	0		
OCD+ Drug Abuse	1 (6.7)	0	0		
None	11 (73.3)	12 (70.6)	11 (61.1)		
Psychiatric drug use, number (%)**	5 (29.41)	4 (26.67)	4 (22.22)	Value=0.24	0.89

*ANOVA, **Chi-square test, ^***^In Iran, facilities are typically concentrated in provincial capitals, while smaller cities have fewer resources.

### 3.2 Time spenditure of clinicians

Therapists’ time allocation for various tasks was documented: On average, the researchers (N. M. and Z. D.) spent approximately 10 hours on patient assessments, totaling 20 hours collectively. Additionally, the researchers and two other therapists each dedicated around 5 hours to training and coordination, resulting in a cumulative 50 hours. Each therapist provided approximately 38 hours of face-to-face treatment, amounting to a total of 152 hours. The principal researcher (N. M.) spent an estimated 4 hours per patient on internet-based treatment, totaling 60 hours for 15 patients.

### 3.3 Adherence to treatment

Adherence to the internet-based treatment was evaluated. A total of 72.8% of participants completed the treatment and submitted all worksheets to the therapist. Additionally, 13.6% of participants completed less than half of the treatment, while 9% completed more than half but ultimately dropped out.

Session attendance or completion served as the primary indicator of adherence. In the face-to-face treatment group, 77.3% of participants completed the treatment and attended all 12 therapy sessions. Meanwhile, 9.1% attended fewer than half of the sessions before dropping out, while 13.6% attended more than half but did not complete the program.

### 3.4 Outcomes

#### 3.4.1 Descriptive findings and statistical assumptions

**Table 4** presents the descriptive findings of the research variables across the three measurement phases, categorized by group. The results indicated that the mean scores of the experimental groups changed at the post-test and follow-up phases. ANOVA assumptions were examined (64), including the absence of significant outliers within groups and the normal distribution of the dependent variable across the groups, assessed using the Shapiro-Wilk test. The test statistic values for all variables were 0.98 or 0.97, with significance level exceeding 0.05, confirming data normality. The assumption of sphericity was assessed and validated using the Mauchly’s test. If the significance level was below 0.05 and the assumption of sphericity was violated, the Greenhouse-Geiser correction was applied.

**Table 4:** Descriptive findings of research variables.

Variables	Phase	Internet-based group		Face-to-face group		Control group	
		Mean	Standard Deviation	Mean	Standard Deviation	Mean	Standard Deviation
Anxiety	Pre-test	42.18	14.10	39.13	16.67	56.44	15.76
	Post-test	30.65	6.18	24.67	16.68	61.00	14.78
	Follow-up	36.94	11.42	27.33	15.15	64.00	15.67
Depression	Pre-test	11.59	3.61	10.40	4.34	14.56	4.25
	Post-test	7.94	1.85	6.80	4.38	16.67	3.99
	Follow-up	9.94	3.33	7.60	3.96	17.33	4.12
Life interference	Pre-test	40.07	22.81	34.18	14.48	47.89	19.95
	Post-test	28.00	16.71	25.76	8.94	50.22	17.02
	Follow-up	27.13	18.29	33.59	13.66	52.67	16.29
Positive affect	Pre-test	30.80	7.20	33.76	6.31	30.72	7.74
	Post-test	32.33	7.63	32.06	5.67	27.17	7.84
	Follow-up	30.73	6.71	28.59	6.18	25.72	7.73
Negative affect	Pre-test	24.53	6.25	28.41	3.78	30.44	7.00
	Post-test	19.33	6.03	22.18	2.94	30.72	8.83
	Follow-up	20.67	5.07	24.12	5.83	31.67	10.28
Emotional avoidance strategy	Pre-test	62.82	12.80	67.07	16.39	69.39	14.65
	Post-test	51.12	8.55	52.53	14.10	68.72	15.71
	Follow-up	57.12	10.09	55.00	11.54	69.67	12.72
Distress tolerance	Pre-test	44.73	9.60	44.76	5.71	38.17	10.08
	Post-test	49.80	11.32	48.24	8.70	39.94	10.64
	Follow-up	47.07	11.45	47.82	7.00	41.44	9.93

#### 3.4.2 Pairwise comparisons

**Tables 5**, **6** present the results of pairwise comparison (Bonferroni-adjusted) of groups across the three measurement phases within each group. Given the small sample size, these results should be interpreted as preliminary and warrant further investigation with larger samples.

**Table 5:** Results of pairwise comparison of groups by three stages of measurement.

Variable	Group	Phase	Phase	Mean difference	Standard deviation	Level of significance
Anxiety	Face-to-face group	Pre-test	Post-test	14.47	3.69	**<0.001**
		Post-test	Follow-up	-2.67	1.99	0.56
	Internet-based group	Pre-test	Post-test	11.53	3.47	**0.005**
		Post-test	Follow-up	-6.29	1.87	**0.005**
	Control group	Pre-test	Post-test	-4.56	3.37	0.55
		Post-test	Follow-up	-3.00	1.82	0.32
Depression	Face-to-face group	Pre-test	Post-test	3.60	0.95	**0.001**
		Post-test	Follow-up	-0.80	0.53	0.40
	Internet-based group	Pre-test	Post-test	3.65	0.89	**<0.001**
		Post-test	Follow-up	-2.00	0.49	**<0.001**
	Control group	Pre-test	Post-test	-2.11	0.87	0.57
		Post-test	Follow-up	-0.67	0.48	0.51
Life interference	Face-to-face group	Pre-test	Post-test	8.41	4.01	**0.04**
		Post-test	Follow-up	-7.82	2.22	**<0.001**
	Internet-based group	Pre-test	Post-test	12.07	4.26	**0.01**
		Post-test	Follow-up	0.87	2.37	0.72
	Control group	Pre-test	Post-test	-2.33	3.89	0.55
		Post-test	Follow-up	-2.44	2.16	0.26
Negative affect	Face-to-face group	Pre-test	Post-test	6.24	1.80	**0.00**
		Post-test	Follow-up	-1.94	1.16	0.31
	Internet-based group	Pre-test	Post-test	5.20	1.92	**0.03**
		Post-test	Follow-up	-1.33	1.24	0.86
	Control group	Pre-test	Post-test	-0.28	1.75	1.00
		Post-test	Follow-up	-0.94	1.13	1.00
Positive affect	Face-to-face group	Pre-test	Post-test	1.71	1.63	0.90
		Post-test	Follow-up	3.47	1.05	**0.01**
	Internet-based group	Pre-test	Post-test	-1.47	1.73	1.00
		Post-test	Follow-up	1.60	1.12	0.48
	Control group	Pre-test	Post-test	3.56	1.58	0.09
		Post-test	Follow-up	1.44	1.02	0.49
Emotional avoidance strategy	Face-to-face group	Pre-test	Post-test	14.53	3.67	**<0.001**
		Post-test	Follow-up	-2.47	1.91	0.61
	Internet-based group	Pre-test	Post-test	11.71	3.44	**0.00**
		Post-test	Follow-up	-6.00	1.79	**0.00**
	Control group	Pre-test	Post-test	0.67	3.35	1.00
		Post-test	Follow-up	-0.94	1.74	1.00
Distress tolerance	Face-to-face group	Pre-test	Post-test	-3.47	2.57	0.55
		Post-test	Follow-up	0.41	2.68	1.00
	Internet-based group	Pre-test	Post-test	-5.07	2.73	0.21
		Post-test	Follow-up	2.73	2.85	1.00
	Control group	Pre-test	Post-test	-1.78	2.50	1.00
		Post-test	Follow-up	-1.50	2.60	1.00

Adjustments for multiple comparisons were made using the Bonferroni correction. Bold numbers indicate significant differences.

**Table 6:** The results of the pairwise comparison of the three stages of variable measurement by groups.

Variable	Phase	Group	Group	Mean difference	Standard deviation	Level of significance
Anxiety	Post-test	Face-to-face group	Internet-based group	-5.98	4.69	0.62
		Face-to-face group	Control group	-36.33	4.62	**<0.001**
		Internet-based group	Control group	-30.35	4.47	**<0.001**
	Follow-up	Face-to-face group	Internet-based group	-9.61	5.03	0.19
		Face-to-face group	Control group	-36.67	4.96	**<0.001**
		Internet-based group	Control group	-27.06	4.80	**<0.001**
Depression	Post-test	Face-to-face group	Internet-based group	1.14	1.26	1.00
		Face-to-face group	Control group	-9.87	1.24	**<0.001**
		Internet-based group	Control group	-8.73	1.20	**<0.001**
	Follow-up	Face-to-face group	Internet-based group	-2.34	1.35	0.27
		Face-to-face group	Control group	-9.73	1.33	**<0.001**
		Internet-based group	Control group	-7.39	1.29	**<0.001**
Life interference	Post-test	Face-to-face group	Internet-based group	-2.24	5.20	0.67
		Face-to-face group	Control group	-24.46	4.96	**<0.001**
		Internet-based group	Control group	-22.22	5.13	**<0.001**
	Follow-up	Face-to-face group	Internet-based group	6.46	5.70	0.26
		Face-to-face group	Control group	-19.08	5.44	**<0.001**
		Internet-based group	Control group	-25.53	5.62	**<0.001**
Negative affect	Post-test	Face-to-face group	Internet-based group	2.84	2.30	0.67
		Face-to-face group	Control group	-8.55	2.19	**<0.001**
		Internet-based group	Control group	-11.39	2.27	**<0.001**
	Follow-up	Face-to-face group	Internet-based group	3.45	2.69	0.62
		Face-to-face group	Control group	-7.55	2.56	**0.02**
		Internet-based group	Control group	-11/00	2.65	**<0.001**
Positive affect	Post-test	Face-to-face group	Internet-based group	-0.28	2.52	1.00
		Face-to-face group	Control group	4.89	2.40	0.14
		Internet-based group	Control group	5.17	2.49	0.13
	Follow-up	Face-to-face group	Internet-based group	-2.15	2.46	1.00
		Face-to-face group	Control group	2.87	2.34	0.68
		Internet-based group	Control group	5.01	2.42	0.13
Emotional avoidance strategy	Post-test	Face-to-face group	Internet-based group	1.42	4.67	1.00
		Face-to-face group	Control group	-16.19	4.60	**0.00**
		Internet-based group	Control group	17.61	4.45	**<0.001**
	Follow-up	Face-to-face group	Internet-based group	-2.12	4.08	1.00
		Face-to-face group	Control group	-14.67	4.03	**0.00**
		Internet-based group	Control group	-12.55	3.90	**0.00**
Distress tolerance	Post-test	Face-to-face group	Internet-based group	-1.57	3.63	1.00
		Face-to-face group	Control group	8.29	3.46	0.06
		Internet-based group	Control group	9.86	3.58	**0.03**
	Follow-up	Face-to-face group	Internet-based group	-0.76	3.39	1.00
		Face-to-face group	Control group	6.38	3.23	0.16
		Internet-based group	Control group	5.62	3.34	0.30

Adjustments for multiple comparisons were made using the Bonferroni correction. Bold numbers indicate significant differences.

The review of the tables indicates that, in the face-to-face treatment group, levels of anxiety (p < 0.001), depression (p < 0.001), functional interference (p = 0.04), negative affect (p = 0), and emotional avoidance strategies (p < 0.001) significantly decreased at the post-test compared to pre-test scores. Additionally, anxiety (p < 0.001), depression (p < 0.001), functional interference (p < 0.001), negative affect (p < 0.001), and emotional avoidance strategies (p = 0) in the face-to-face treatment group were significantly lower at the post-test compared to the control group.

Similarly, in the internet-based treatment group, levels of anxiety (p = 0.005), depression (p < 0.001), functional interference (p = 0.01), negative affect (p = 0.03), and emotional avoidance strategies (p = 0) significantly decreased at the post-test compared to pre-test scores. Additionally, anxiety (p < 0.001), depression (p < 0.001), functional interference (p < 0.001), negative affect (p < 0.001), and emotional avoidance strategies (p < 0.001) were significantly lower at the post-test compared to the control group.

At the six-month follow-up, a significant difference remained between the control and the face-to-face treatment group in anxiety (p < 0.001), depression (p < 0.001), functional interference (p < 0.001), negative affect (p = 0.02), and emotional avoidance strategies (p = 0). However, in the face-to-face group, levels of anxiety (p = 0.56), depression (p = 0.40), functional interference (p < 0.001), negative affect (p = 0.31), and emotional avoidance strategies (p < 0.001) increased six months after treatment ended. In contrast, levels of anxiety (p < 0.001), depression (p < 0.001), functional interference (p < 0.001), negative affect (p < 0.001), and emotional avoidance (p = 0) in the internet-based treatment group remained significantly lower than those in the control group (p < 0.001).

Following treatment, the internet-based treatment group exhibited lower levels of depression (p = 1.00), functional interference (p = 0.67), negative affect (p = 0.67), and emotional avoidance strategies (p = 1) compared to the face-to-face treatment group. However, these differences were not statistically significant. At the six-month follow-up, the internet-based treatment group continued to display lower levels of depression (p = 1.00), functional interference (p = 0.26), negative affect (p = 0.62) and emotional avoidance (p = 1) than the face-to-face treatment group, though these differences remained statistically insignificant.

A significant main effect of group was observed for anxiety, F(2, 47) = 28.68, p< 0.001, ƞ^2^p = 0.55, depression F(2, 47) = 28.33, p < 0.001, ƞ^2^p = 0.55, and functional interference, F(2, 47) = 9.88, p = 0, ƞ^2^p = 0.30. A significant within-subjects effect of time was identified for anxiety, F(2.80, 65.64) = 6.91, p < 0.001, ƞ^2^p = 0.23, depression F(2.70, 63.32) = 9.57, p = < 0.001, ƞ^2^p = 0.29 and functional interference, F(2.86, 66.97) = 3.84, p = 0.15, ƞ^2^p = 0.14.

In the face-to-face treatment group, positive affect (p = 0.14) and distress tolerance (p = 0.06) at post-test did not significantly differ from the control group. In the internet-based treatment group, positive affect (p = 1) and distress tolerance (p = 0.21) did not show significant changes at post-test compared to either pre-test scores or the control group. At the six-month follow-up, no significant differences were observed between the control group and the face-to-face treatment group in positive affect (p = 0.68) and distress tolerance (p = 0.06).

At follow-up, positive affect (p = 0.13) and distress tolerance (p = 0.30) in the internet-based treatment group did not significantly differ from those in the control group. After treatment completion, no significant differences were found in positive affect (p = 0.13) and distress tolerance (p = 1) between the internet-based and the face-to-face treatment groups. Although the internet-based treatment group exhibited higher levels of positive affect (p = 1) and distress tolerance (p = 1) than the face-to-face treatment group at follow-up, this differences were not statistically significant. Eleven participants in the control group remained in treatment after the study was completed, and of these, seven chose internet-based and four chose face-to-face treatment.

At post-test scores for anxiety, the face-to-face treatment group demonstrated significantly greater improvement compared to the control group (Hedges’ g = 2.32, 95% Cl [1.43, 3.20], indicating a large effect size). The internet-based treatment group also demonstrated significantly greater improvement compared to the control group (Hedges’ g = 2.65, 95% Cl [1.74, 3.56], indicating a large effect size). However, the difference between face-to-face and internet-based treatments was medium (Hedges’ g = -0.49, 95% Cl [-0.22, 1.19]) At follow-up, the face-to-face treatment group showed a small effect compared to the control group ((Hedges’ g = 2.38, 95% Cl [1.48, 3.27]). The internet-based treatment group also had a large effect compared to the control group (Hedges’ g = 1.97, 95% Cl [1.16 - 2.77]). However, the difference between face-to-face and internet-based treatments was medium (Hedges’ g = -0.72, 95% Cl [-1.44, 0]).

At post-test scores for depression, the face-to-face treatment group exhibited significantly greater improvement relative to the control group (Hedges’ g = 2.37, 95% Cl [1.47, 3.26], suggesting a large effect size). Similarly, the internet-based treatment group also showed substantial improvement compared to the control group (Hedges’ g = 2.78, 95% Cl [1.85, 3.70]). However, the difference between face-to-face and internet-based treatments was of small magnitude (Hedges’ g = 0.35, 95% Cl [-0.35, 1.05]). At follow-up, the face-to-face treatment group showed a large effect compared to the control group ((Hedges’ g = 2.40, 95% Cl [1.51, 3.30]). The internet-based treatment group also had a large effect compared to the control group (Hedges’ g = 1.96, 95% Cl [1.15 - 2.77]). However, the difference between face-to-face and internet-based treatments remained at a medium level (Hedges’ g = -0.64, 95% Cl [-0.07, 1.36]).

In the post-test assessment of functional interference, the face-to-face treatment group demonstrated significantly greater improvement compared to the control group, suggesting a large effect size (Hedges’ g = 1.78, 95% Cl [1, 2.57]). Similarly, the internet-based treatment group exhibited notable improvement relative to the control group (Hedges’ g = 1.32, 95% Cl [0.56, 2.07]). However, the difference in effectiveness between face-to-face and internet-based treatments was of small magnitude (Hedges’ g = 0.17, 95% Cl [-0.53, 0.87]). At follow-up, the face-to-face treatment group continued to show a large effect compared to the control group ((Hedges’ g = 1.27, 95% Cl [0.54, 1.99]). The internet-based treatment group similarly maintained a large effect in comparison to the control group (Hedges’ g = 0.85, 95% Cl [0.71, 2.26]). The difference between the two treatment modalities remained small (Hedges’ g = -0.40, 95% Cl [-1.11 - 0.30]).

**Figure 5:**
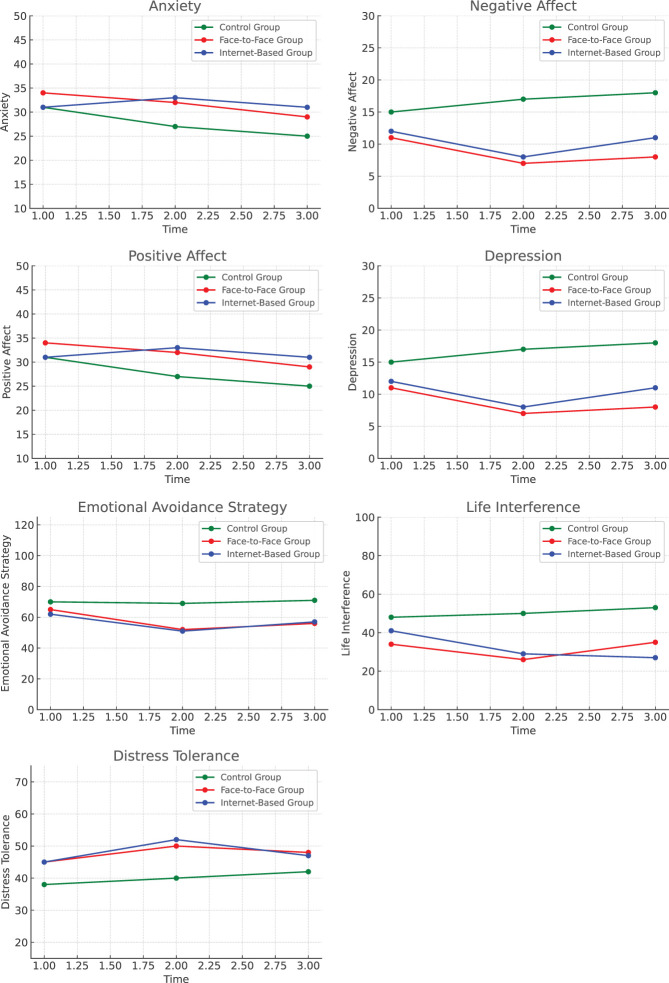
Diagram of therapeutic interventions.

**Figure 5** Presents a diagram of therapeutic interventions.

## 4 Discussion

This pilot study aimed to evaluate the efficacy of face-to-face and internet-based unified transdiagnostic treatments in reducing symptoms and improving functioning in adolescents with anxiety and depressive disorders. The results indicate that both modalities effectively reduced anxiety and depression, showing a significant difference from the control group. However, no significant differences were observed between the two treatment types and neither was superior to the other, in all the primary and secondary outcomes. The Internet-based treatment maintained its effectiveness up to six months post-treatment, whereas this was not the case for face-to-face treatment. Additionally, both treatment formats successfully reduced negative affect and emotional avoidance but did not significantly improve positive affect or distress tolerance. The fact that mean anxiety and depression levels for participants in both treatment groups fell below the clinical cutoff after treatment and remained low during the follow-up highlights the clinical significance of these interventions. However, due to the limited sample size, these findings should be interpreted with caution. Future studies with larger populations are needed to confirm these results. While preliminary, these findings are crucial in shaping future research, generalizations should be made cautiously given the small participant pool.

These promising effects align with the UP-A findings reported by the protocol drafting group and previous research. The core modules of UP-A emphasize adolescents’ negative responses to emotions and emotional avoidance (65–71), which are fundamental transdiagnostic processes contributing to emotional disorder vulnerability and maintenance s (65–71). Additionally, these findings support systematic reviews and meta-analyses suggesting that internet-based psychotherapies are as effective as face-to-face treatments for reducing anxiety and depression (40, 72–74). One potential explanation for the comparable efficacy of internet-based transdiagnostic psychotherapy may be the presence of a therapist guiding adolescents throughout the treatment, as along with parental involvement through a parent-related pamphlet (75).

Additionally, the results showed that treatment effects in the Internet-based treatment group were sustained up to the six-month follow-up. This may be attributed to the transdiagnostic nature of the treatment. For example, adolescents learn new strategies to manage situations and cognitions that trigger intense emotions and continued practicing them over time (69). In contrast, the face-to-face treatment group did not maintained these effects. Similar findings have been reported in reviews of remote therapies for adolescents, which showed a combined effect size of 0.44- comparable to or slightly higher than that of face-to-face psychotherapies (76). Although changes over time cannot be entirely attributed to treatment effects, one possible reason for the superior long-term performance of internet-based treatment is that adolescents may remain engaged with therapeutic content even after completing treatment. Since therapy content remains accessible offline, which was confirmed by website usage data in this study. Additionally, learning and practicing therapeutic skills at home rather than in a clinical setting may facilitate greater integration of these skills into daily life for adolescents and their families. Another contributing factor to these outcomes may be the flexibility of the internet-based treatment. In this study, participants in the internet-based group had the freedom to decide when to access the website, often resulting in longer treatment durations. In contrast, face-to-face treatment followed a fixed schedule set by therapists. Furthermore, this study utilized a six-month follow-up period, whereas most studies in this field typically assess outcomes after only three months.

The limited effectiveness of both face-to-face and internet-based treatments in improving positive affect and distress tolerance may be due to treatment protocols primarily targeting the reduction of negative affect rather than the enhancement of positive affect. Furthermore, increasing positive affect in adolescent psychotherapy presents unique challenges. Adolescents undergo significant developmental changes that influence mood and emotion regulation. The brain regions responsible for emotion regulation are still maturing, making it difficult for adolescents to sustain positive affect consistently. Additionally, adolescents are highly influenced by their environment, including family dynamics, peer relationships, and academic stress. Negative experiences in these areas can contribute to a declines in positive affect (77). Cultural differences also play a significant role. In Western culture, individuals tend to maximize positive affect and minimize negative affect, as positive emotions are associated with independence and self-reliance, while negative emotions are less accepted. In contrast, East Asian cultures favor moderate levels of positive affect and tolerate low levels of negative affect, recognizing both the drawbacks of positive emotions and the benefits of negative emotions (78, 79). The rise in boredom and anhedonia, along with decreased positive affect among adolescents- particularly girls- become evident following the COVID-19 pandemic. As a result of physical distancing measures, social rewards such as attending school and participating in sports were reduced, leading to lasting negative psychological effects that persisted even after restrictions were lifted (80–83).

Considering the efficacy of treatment on other transdiagnostic variables (excluding positive affect) and that the findings of previous research demonstrating increased distress tolerance with transdiagnostic protocols, it is possible that “Distress Tolerance Scale” questions were not clearly understood by most adolescents. For example, statements such as “my feelings of distress or agitation are unacceptable” or “I do anything to stop my feelings of distress or agitation” may not have been fully comprehended. This assumption was supported by a review of the answer sheets, which revealed inconsistencies in participant responses. Furthermore, as shown in **Figure 5**, even before treatment, participants had relatively high scores on this scale. The average scores across all three groups were higher than those typically observed in distressed adolescents, suggesting potential issues with comprehension of the scale items.

### 4.1 Limitations and recommendations

Despite the promising results, this study had several limitations. The most significant limitation was the small sample size, coupled with a high dropout rate and an underrepresentation of male participants. Additionally, resource constraints limited the number of cities included in the study. All results were based on self-report scales, which may have influenced the treatment outcomes. To minimize bias in treatment implementation, improve research reliability, and address the time demands of face-to-face treatment, four designate therapists conducted the treatment sessions. However, treatment outcomes may have varied depending on therapist -specific factors, such as their ability to establish therapeutic rapport, implement intervention guidelines, facilitate sessions, classify problems, resolve treatment issues, and respond sensitively to patient needs (84, 85). Thus, while the use of four therapists ensured consistency, it also introduced variability that could be considered both a strength and a limitation of the study.

Accordingly, future research on adolescent transdiagnostic protocols should examine both face-to-face and internet-based treatments with larger, more diverse samples, particularly including more adolescent boys and participants from different cities and rural areas. Additionally, future studies should differentiate between evaluators and therapists during the clinical process and incorporate alternative assessment methods, such as clinical interviews, to enhance measurement reliability. Future research is needed to assess the effectiveness of the Internet-based protocol for other emotional disorders. Moreover, standardized instruments specifically designed for adolescents should be used to measure distress tolerance, rather than relying on the current distress tolerance scale. Lastly, process-oriented research is essential to better understand the underlying mechanisms of Internet-based transdiagnostic treatment for adolescents.

One limitation of this study is the absence of a previously published study protocol. While the study design followed established methodological guidelines, the lack of a publicly available protocol may limit reproducibility and transparency.

### 4.2 Conclusion

This study is the first in Iran to compare the efficacy of face-to-face and Internet-based transdiagnostic psychotherapy for adolescents with anxiety and depressive disorders. The findings indicate that while both treatment modalities effectively reduced anxiety and depression symptoms and improved adolescents functioning, no significant different was observed between the two treatment groups. However, both treatments demonstrated a significant advantage over the control group. Furthermore, the results suggest that both face-to-face and internet-based transdiagnostic treatments successfully addressed key transdiagnostic processes, such as emotional avoidance and negative affect, in adolescents with anxiety and depressive disorders. Nonetheless, neither treatment significantly improved positive affect or distress tolerance.

Adolescent psychologists should acknowledge the advantages of the transdiagnostic approach in treating emotional disorders. While treatment should be tailored to each individual’s experiences, it must also comprehensively target the core mechanisms underlying these disorders. Additionally, the Unified Protocol for Adolescents (UP-A) offers a cost-effective and time-efficient intervention. In situations where evidence-based face-to-face treatment is inaccessible, this approach presents a viable alternative, with the potential to sustain its efficacy over time- possibly even surpassing face-to-face treatment. Given the significant barriers to accessing evidence-based psychotherapy, particularly in low- and middle-income countries like Iran, future research should rigorously evaluate the long–term effectiveness of these interventions.

## Data Availability

The raw data supporting the conclusions of this article will be made available by the authors, without undue reservation.
